# Inclusive Growth in the Era of Automation and AI: How Can Taxation Help?

**DOI:** 10.3389/frai.2022.867832

**Published:** 2022-05-31

**Authors:** Rossana Merola

**Affiliations:** International Labour Organization (ILO), Research Department, Geneva, Switzerland

**Keywords:** robot tax, digital tax, automation, artificial intelligence, tax policy, inequality, technological unemployment

## Abstract

In the last decades, the world economy is facing a massive rise in automation, robotics and Artificial Intelligence (AI) which, according to some analysts, could lead to significant job losses or job polarization and hence widen income and wealth disparities. This scenario may impede the achievement of the Sustainable Development Goal 8 (SDG 8). In this context, the role of government and regulation becomes crucial in order to prevent an undesirable scenario, where technological change, namely automation and AI, comes at the cost of mass unemployment and growing inequality. This paper focuses on the role of taxation as a possible tool for sharing the gains from automation and AI. Nowadays, advances in technology may have a direct impact on tax systems, which should be re-adapted to take into account new forms of jobs and new business models. The paper discusses pros and cons of several possible solutions and then compares progresses achieved in different countries. Concerning robot tax and digital taxes there are already some concrete steps undertaken both at national and international level, while other proposals remain still nebulous. Of course, taxation *per se*, and any single policy in general, is not sufficient to achieve a more inclusive and equal growth. It is instead crucial to create synergies across policies and a strong link between employment creation strategies, redistributive policies, skill development and social protection systems.

## Introduction

In the last decades, the world economy has witnessed a massive process of automation, robotization and artificial intelligence (AI), which can already replace humans in a range of activities. Advanced robotics, machine learning and AI already find diverse applications, including digital assistants such as the Google Assistant or Siri, speech and image recognition, text translation and automatic text generation. More sophisticated applications include medical systems for diagnosis of pathologies (medtech), automated review of legal contracts (lawtech), self-driving cars, the detection of patterns in stock markets for successful trading (algorithmic trading) and the estimation of building's interior temperature (Villa and Sassanelli, 2020).

Many analysts are rising concerns about the risk that advances in robotization and AI may lead to significant job losses or job polarization and ultimately result in widening income and wealth disparities (Méda, 2016; Korinek and Stiglitz, 2017). Among these, Frey and Osborne (2017) find that over the next 20 years technology may displace a large share of human workers, precisely 35% in the United Kingdom and 47% in the United States. In a report published in 2018,^1^ the World Economic Forum warned that by 2025 more than 50% of current jobs will be automated. Jobs in Eastern and Southern Europe, Germany, Chili and Japan are more automatable than those in Anglo-Saxon and Nordic countries (Nedelkoska and Quintini, 2018). While some studies cast doubts on the job loss effect of technology in advanced economies, there is consensus on the effects in emerging economies which rely more on manufactory and are facing robot-driven reshoring (see Carbonero et al., 2018; De Backer et al., 2018). According to the World Bank (2016), the risk of job loss in developing countries is even higher than in advanced economies: 69% in India, 72% in Thailand, 77% in China, and a massive 85% in Ethiopia.^2^

Job losses due to automation are likely to widen inequality. According to the common view, automation is likely to penalize medium-skilled workers more than low- and high-skilled workers, as their tasks can be more easily replaced by AI and robots. Many commenters have hence argued that technological progress should not come at the expense of more vulnerable people and that solving inequity should be a priority for governments. However, decreasing labor income could create limitations for governments in the use of labor taxation as a tool for redistributing wealth, which further exacerbates inequality.

This scenario threatens the achievement of Sustainable Development Goal 8 (SDG 8) in the United Nations 2030 Agenda for Sustainable Development. SDG 8 exhorts the international community to “promote sustained, inclusive and sustainable economic growth, full and productive employment and decent work for all”.

It is evident that while technological progress certainly improves life quality, it may nevertheless produce serious social, economic and political harms if it remains unregulated (Acemoglu, 2021). In light of this context, the role of governments and regulation becomes crucial in order to prevent an undesirable scenario, where technological change comes at the cost of mass unemployment and growing inequality. Therefore, governments and enterprises should take steps to preserve competition and avoid monopolistic power, updating skills and redistribute profits.

This paper focuses only on the role of taxation as a possible tool for sharing the gains from automation and AI. The aim is to shed light on possible solutions, being aware that each of them presents strengths and weaknesses. Nowadays, technological progress is radically changing the society and may have a direct impact on tax systems, which should be re-adapted to take into account new forms of jobs and new business models. Section Challenges Arising From Robots and Artificial Intelligence summarizes the discussion in the policy debate on the possible effects of robots and AI on employment and inequality. The lack of agreement makes policy interventions even more relevant in order to minimize possible negative effects of technological change and to make sure that gains from robotization and AI are equally shared. Section Tax Solutions presents several tax policy solutions, discusses pros and cons of each of them and then compares progresses achieved in different countries and at international level. Concerning the robot tax and the digital tax, there are already some concrete steps undertaken by some governments in advanced economies, while other proposals remain more nebulous. Finally, Section Conclusions concludes.

Of course, taxation *per se*, and any single policy in general, is not sufficient to achieve a more inclusive and equal growth. It is instead crucial to create synergies across policies and a strong link between employment creation strategies, redistributive policies, skill development and social protection systems.

## Challenges Arising From Robots and Artificial Intelligence

The widespread adoption of AI poses several challenges, related to modalities of consumers' data collection which are often intrusive and not transparent, privacy protection and cyber-security in e-commerce (D'Adamo et al., 2021; Puntoni et al., 2021).

This section focuses on challenges for labor market and equity. Despite some afore-mentioned studies are warning that technological progress may cause job losses and widening inequality, so far there is no agreement in the literature on the effects of robotization and AI on employment and inequality.

According to some studies, employment effects specifically from adopting robots remain rather limited or are even positive at aggregate level. Among these, Dixon et al. (2021) compare employment and performance outcome between robot-adopting and non-adopting firms in Canada. They find that employment increases in robot-adopting firms, especially for low-skilled workers. Similar results are found by Acemoglu et al. (2020) for French manufacturing firms and by Koch et al. (2019) for Spanish firms. These studies also find an increase in performance (i.e., TFP or total revenues) in those firms adopting robots. Using data on US textile, steel and auto industries, Bessen (2017) argues that technological progress may at the same time be beneficial for some industries and hit some others. Ryan Avent, an editor and columnist for *The Economist*, points out that employment remains very high in many advanced countries, such as Germany and Japan, although they make an intense use of robots.

Looking more specifically at AI, the final effect on employment will be determined by the coexistence of three effects: task-substitution, task-complementarity and creation of new jobs. In the case of matching applications (e.g., Linkedin, Amazon), algorithms are already used to match supply and demand and hence easily replace human workers. In the case of classification/screening tasks, AI can assist workers but without substituting them. An example might be computer-assisted surgery which allows surgeons to perform surgical intervention remotely. In this case, there is no substitution, but a kind of “cobotisation”, that is a co-working between humans and artificial intelligence, which can ultimately increase overall productivity. Finally, concerning process-management tasks, AI can perform tasks that human workers are not capable to perform. Moreover, the digital economy has created new types of jobs (e.g., AI-programmers, e-commerce specialists, apps and software developers, crowd-workers, influencers and those working on social media).

Keeping this in mind and considering the scarcity of data and difficulties in measuring the exposure to AI, it is difficult nowadays to predict the overall effect of AI and automation on jobs. The final effect will depend on which effect will dominate. Georgieff and Hyee (2021) find that task substitution dominates only for workers with low digital skills, while productivity effects dominate for workers with good digital skills. In addition, the final effect also depends on the adaptability of jobs in the digital transformation (Arntz et al., 2017). In this light, some studies share an optimistic view. Brynjolfsson et al. (2018) state that digitalization could also lead to reorganization of occupations rather than replacement. In a similar vein, Bessen (2017) argue that “automation might not cause mass unemployment, but it may well require workers to make disruptive transitions to new industries, requiring new skills and occupations”.

Concerning the effects on inequality, new technologies in the last years have been associated to greater inequality and job polarization. Automation due to AI, robots and computers is likely to affect mostly middle-class jobs. Humans are already being replaced, partially or fully, in some tasks as legal services, accounting, logistics and retail. Displaced workers are likely to compete downwards, rather than falling into unemployment. This scenario suggests further job polarization in the next years. However, according to a recent study by Michael Webb, while robots and software may take over middle-skilled tasks, AI may perform high-skilled tasks and hence is expected to have the reverse effect on inequality, since better-educated and better-paid workers will be the most affected by the new AI-based technologies. Still, this study warned that while AI will reduce 90:10 wage inequality, it will not have an impact on the top 1% earners.

Inequality has increased not only across workers undertaking different tasks in the same firm, but also across firms. According to recent research conducted at the World Bank by Kelly et al. (2017), at least in Europe, the main driver of wage inequality is the wage gap across firms, which is determined by differences in the rate of adoption of digital technologies. As pointed out by Ernst (2019), in this era of AI, we are witnessing the emergence of a new business model, called “surveillance capitalism”, which is based on collecting data without barriers to access and exploited with proprietary algorithms. While the data come free—and users are often all too willing to give up their privacy—data collection is not since it is protected by intellectual property rights. While on the one side the rise of new “big data” platforms, able to collect huge information on consumer behaviors and preferences, can certainly improve the efficiency in the economy, on the other side “big data” have encouraged the emergence of “superstar” firms which are outperforming compared to the other firms in the economy. These “superstars”, mostly digital companies such as Facebook, Google, Amazon and Netflix, collect huge amounts of data which allow them to individualize prices and product offers and cumulate profits and wealth. “Superstar” firms are then able to gain market power and not surprisingly, concentrated winner-take-all markets are associated with the fall in the labor share (see Autor et al., 2017; Barkai, 2020).

These different forms of inequalities require different forms of tax interventions. We will discuss different alternative tax policies in the following sections. One of the main arguments in favor of a tax on robots is that it preserves low-skill jobs which are more likely to be automated. In this regard, a robot tax can address inequality caused by skill-biased technological change. Another option could be wage subsidies for low-skilled workers. However, inequality may also arise because the emergence of a new business model, called “surveillance capitalism”, concentrates profits and wealth in the hands of few “superstars” firms, mostly digital companies. In this case, other types of taxes would be preferable, such as digital taxation, new tax on corporations' stock shares or the creation of sovereign funds. In particular, the latter two solution are deemed to be progressive since stock ownership is highly concentrated among the richest.

To achieve more inclusive and equal growth, taxation should go hand in hand with other type of policies. Digital businesses can easily collect a huge amount of data from their users. Governments and private businesses should acknowledge that users' data represent an incredibly valuable source of profits and take steps to ensure that markets remain contestable and competitive. On the one side, there are proposals to tax the income or rents generated by the exploitation of users' data.^3^ On the other side, there are proposals to share data for free in order to guarantee market competition. In a recent article, Ekkehard Ernst discussed several solutions to address potential rise in inequality in the era of “surveillance capitalism”.^4^ Considering data as a common good which allows the extraction of rents would help restore the balance between individual data suppliers and corporate platform providers. Treating data ownership as a collective-action problem can limit the increases in concentration and market power and will ultimately help to address the continuous rise in inequality. Moreover, it is crucial that both governments and enterprises support the existing workforces through reskilling and upskilling. Governments should implement effective policies to facilitate the transition to the new world of work where humans will co-work with artificial intelligence, without leaving anybody behind. In this light, a necessary step is readapting the current education system to support the transit to new tasks required by AI-based technologies.

## Tax Solutions

This section discusses different tax solutions which can ensure that gains from AI and technological changes are equally shared. Some proposals are already on track, while others remain more nebulous and/or limited to a few countries. Each proposal presents both strengths and weakness.

### Robot Tax

The most immediate solution, which has been strongly supported among others by Bill Gates, Elon Musk and Nobel Laureate in Economics Robert Shiller, is taxing robots. A robot tax stems from the idea that robot-adopting firms should pay a tax since they replace human workers with robots. There are several arguments in favor of robot tax. The first one is preserving human employment by introducing disincentives for firms from replacing humans with robots. Second, even though firms prefer replacing humans with robots, a robot tax would generate revenues for the government to cover the loss of revenues from payroll taxes and income tax.^5^ A third argument in favor of the robot tax is allocation efficiency: robots do not pay neither payroll taxes, nor income taxes. Taxing robots improves the efficiency in the economy, because governments already tax labor, so they should also tax robots at the same rate to avoid distortion in the resource allocation. In most of advanced economies, and in particular in the United States, taxation favors AI and automation over human employment.^6^ This may distort investment toward automation simply because companies benefit from tax windfalls and not because automation may increase profitability.^7^ Finally, not taxing robots will increase income inequality, because of the decreasing share of national income going to labor.

Revenues from the robot tax can be redistributed as universal basic income or as transfers to workers displaced in their jobs by robotic systems and AI and not able to be relocated in new jobs. New York Mayor, Bill de Blasio, proposed to use revenues from the robot tax to create new jobs in green energy, health care and education.

There are also arguments against the robot tax. First of all, as discussed in Section Challenges Arising From Robots and Artificial Intelligence, according to some studies employment effects from adopting robots remain rather limited or even positive at aggregate level (Acemoglu and Restrepo, 2017; Bessen, 2017; Graetz and Michaels, 2018; Koch et al., 2019; Acemoglu et al., 2020; Dixon et al., 2021).

The main argument against taxing robots, however, is that it might impede innovation in an era of productivity slump. Over the last decades, advanced economies have experienced stagnating productivity. Taxing new technologies could make that slowdown worse, while according to some studies investing in robots enhances growth and productivity. A CEBR (2017) study finds that investment in robots contributed to 10 percent of GDP growth per capita in OECD countries from 1993 to 2016. Graetz and Michaels (2018) find that a unit increase in robotics density (defined as the number of robots per million of hours worked) is associated with a 0.04 percent increase in labor productivity. An analysis carried out by the Institute for Employment Research and the Düsseldorf Institute for Competition Economics finds that from 2004 to 2014 GDP has increased by 0.5% per person per robot as result of robotization (CEBR, 2017).

Finally, another argument against the robot tax is that it would reduce the incentive for companies to invest in innovation and will make low wage traps more persistent, as argued by Robert D. Atkinson, president of the Information Technology and Innovation Foundation (ITIF). According to Atkinson, the main reason behind wage and GDP growth stagnation in advanced economies is the productivity slow-down. As mentioned above, there is empirical evidence that robots are driving labor productivity and GDP growth (CEBR, 2017; Graetz and Michaels, 2018). Therefore, creating disincentives to robotization may further impede labor productivity and perpetuate wage stagnation.

Provided that automation increases overall productivity and efficiency and hence is beneficial to the society, hence the robot tax should be designed so to avoid discouraging the use of robots and automation. Some research shows that it is optimal to tax robots only for a limited time span. In this view, Guerreiro et al. (2020) propose to tax robots for three decades.

Beside the opinion in favor or against the robot tax, there is however still discussion on how companies should pay the robot tax. A first proposal could be to tax robots themselves, in the amount of the salary paid to the hypothetical displaced human worker. This solution is however extremely complicated to be put in practice, since robots are unlikely to replace human workers in the entire set of their tasks. It is more common that robots take over only some tasks previously performed by humans and hence it is quite difficult to find a one-to-one link between the robot and the displaced worker difficult.

Alternatively, another option could be to levy a tax on the use of robots, that is imposing a higher rate of corporate tax for using robots, since companies make higher profits due to the powerful efficiency of robots. This proposal is also complicated to be implemented, because what we see nowadays is a form of “cobotization”, which is a collaboration between robots and human workers to complete a task and jointly contribute to make profits. Therefore, it is not so straightforward to disentangle the profits or value created by the robot from that one created by the human worker.

Another proposal is subjecting robots to VAT, since robots can replace humans in the supply of goods or services which are subject to the VAT. To avoid obstacles to the adoption of new technologies and innovation, a simpler approach could be levying a lump-sum tax, payable at the same level by everyone, which would not create distortions in the economy. However, lump-sum taxes present trade-off in terms of equity and distributional effects to be considered. A lump-sum tax would be regressive and bear more on small businesses. Since every business will pay the same amount of robot tax no matter the profits it runs, absorbing the fixed cost of a robot tax would be more arduous for small family businesses than for large companies.

Overall, these proposals require international coordination to avoid that income could be taxed twice, at the robot level, in the amount of the imputed salary or higher profits associated to the use of robots, and at the corporation level (Oberson, 2017).

Another problem with the robot tax is the definition of robot itself. Some institutions (e.g., the EU Parliament, the International Federation of Robotics) have proposed criteria to define robots.^8^ All definitions include two main criteria: the level of autonomy and the capacity to learn. However, there is still a lack of consensus on the definition of robots. The distinction between a machine and a robot or between a computer program and AI is still not clear. For example, a ticket-vending machine replaces a human but could not be considered a robot.

Moving to more “philosophical” issues, some thorny questions deserve more consideration. First, governments may choose whether to tax robots themselves as they were persons or whether to levy a tax on the use of robots. If they opt for the first solution, then governments should give legal-person status to robots in order to make them taxable, as Professor Xavier Oberson points out. The status of legal person implies that robots would have rights and obligations, so they could collect social security and retire or go to jail if they do not pay taxes.

At this stage, proposals on how to implement a robot tax in practice remain very nebulous. South Korea is the only country to have introduced a kind of robot tax. An extensive talk about the need of a robot tax is starting to emerge in the United Kingdom, the United States, Japan and Canada. We discuss below some country cases in this respect.

South Korea has been the first country to have levied a robot tax on August 6, 2017. Korea is one of the countries with the highest share of robots in the workplace, particularly in the manufacturing industry. However, South Korea has not exactly introduced a tax on individual robots or on the use of robots, rather a reduction in the deductions for increasing automation. Under previous governments, Article 24 of the Restriction of Special Taxation Act established that companies could have between 3 and 7% of their corporate tax deducted, depending on the size of the business. Since August 2017, the new administration of President Moon Jae-in has lowered the tax deduction rate by up to 2% points.

In the United States, New York's Mayor and 2020 presidential candidate, Bill de Blasio, has pointed out the need of to adopt a kind of robot tax to protect those jobs at risk of obsolescence. Revenues from the robot tax might be used to create new jobs in green energy, health care and education. Another example of possible proposal of a robot tax has been put forward by a political candidate in Chicago, Ameya Pawar, who has suggested a two-fold approach: on the one side redeeming subsidies given to companies who do not create the promised number of jobs, and on the other side taxing companies who adopt robots to displace human workers. While calls for a robot tax have emerged in the political debate in the United States, the only concrete example attempting to deal with automation, although a very specific type of automation, is the Autonomous Vehicles Tax Legislation. However, there is not agreement on the definition of “fully autonomous vehicle”. In 2017, the Nevada legislature imposed an excise tax on transportation network companies using fully autonomous vehicles. Similarly, in 2018 the California legislature authorized San Francisco to impose a local tax on transportation network companies using autonomous vehicles. Calls for a similar legislation have emerged in two other states, Massachusetts and Tennessee, but not concrete steps have been taken so far.

In Italy a law proposal in August 2017 suggested to increase the corporate income tax rate by 1% for companies “if the production activity of the company is implemented and managed predominantly from artificial intelligence systems and robotics”. However, no further action has been taken. The proposal presented some pitfalls, in particular the legislation provided neither a definition of “artificial intelligence systems” or “robotics”, nor clear criteria to determine whether a company's activity may be considered “predominantly” implemented and managed by AI or robotics.

In 2017, Ms Mady Delvaux, a member of the European Parliament, tried to introduce a recommendation of a robot tax in a Committee on Legal Affairs Report. However, ultimately the resolution adopted by the European Parliament did not include a robot tax. Although the majority of European leaders agreed on the urgence to control the possible side effects of automation on human employment, the EU was concerned about the risk that a robot tax may impede innovation. In particular Andrus Ansip, the former European Commissioner for Digital Single Market, opposed the robot tax.

There is no large empirical evidence on the effects of the robot tax. In South Korea the introduction of the robot tax is associated with a slow-down in investment in robotics. Koracev (2020) reports that in 2017 the new industrial robot installations in South Korea decreased for the first time since 2012. However, it is difficult to establish with certainty the causality between the reduction in the automation tax credit and the slowdown in robotization.

Conversely, Bogenschneider (2021) reports empirical evidence suggesting that higher taxation does not seem to discourage robotization. The empirical evidence shows that “robot density is positively associated with high corporate tax rates, such as in Germany, Japan, South Korea and the Nordic countries, with little or no automation occurring in tax havens where the value of tax deductions for capital investment is zero”.

### Digital Taxes

Another solution is digital taxation. The debate on digital taxation focuses on two main aspects. First, how to ensure that tax policy remains neutral in targeting traditional and digital businesses? Digital businesses have benefitted from preferential tax regimes, e.g., tax advantage for income earned from intellectual property, shorter amortization for intangibles, R&D tax relief. The risk is that preferences for digitalized businesses may create tax windfalls that can be used in ways that distort investment, rather than focusing on innovation.

Second, digital companies may operate without having physical presence in countries where digital enterprises have customers, since they can reach customers through remote sales and service platforms. The ability of digitalized firms to make profits though cross-border sales without a physical presence poses challenges on the traditional corporate income tax rule. Up to now, digital businesses have paid corporate taxes on profits only in those countries where they had a permanent establishment, so either the headquarter or factory or storefront. This means that the countries where sales are made or where online users are located have no taxing rights over the firm's income.

To tax digital profits, several tools have been considered. A first option consists in extending existing rules. For instance, a country may extend its Value-Added Tax (VAT) and Goods and Services Tax (GST) to include digital services or extend the tax base so to include revenues generated from the provision of digital goods and services. A second option is to levy a Digital Service Tax (DST).

Over the past years, many countries have introduced DST and VAT on digital goods and services at unilateral level, which has highlighted how lack of coordination and alignment of standards may be harmful for the global economy and can potentially lead to economically harmful trade wars. The lack of international coordination over the last years has shed lights on some crucial steps which need to be urgently taken. First of all, the VAT and GST rules need to be revised to ensure that foreign suppliers are accountable for the collection and remittance of these taxes in countries where they sell their goods and services, even without having a physical presence. Lack of coordination may also lead to confusion and impede economic activity, since digital business who sells in different countries where they do not have a permanent establishment need to conform to a large diversity of requirements in each of the countries where they have customers. Moreover, lack of coordination can also facilitate tax avoidance, since multinational enterprises can exploit differences in corporate tax rates. Finally, the risk of double taxation can easily arise, since digital businesses may be taxed twice in the hosting country under the national CIT regime and in the countries where they have customers under the DST.

Countries and international organizations are undertaking various initiatives at national level and more recently also at international level.

Regarding VAT and GST, in most of the OECD countries VAT or GST are levied on a large set of goods and services.^9^

Regarding DST, the situation is more complex. Up to now, digital enterprises have paid corporate income tax in the country where they had a permanent establishment, rather than where consumers or users are located. In practice, a digital enterprise may provide services abroad through digital means without having physical presence abroad and make profits without being subject to corporate income tax in foreign countries. Several countries over the past years have decided to tax digital goods and services and they have unilaterally introduced a DST, which rate was varying across countries.

As of May 2020, Austria, France, Hungary, Italy, Turkey and the United Kingdom have introduced a DST, while a proposal for a DST has been put forward in Spain, the Czech Republic, Slovakia and Poland. Some more timid steps in this direction have been taken in Latvia, Norway and Slovenia. Some cases are discussed more in detail below.^10^

In France in July 2019 a 3% DST has been levied on revenues from digital interface services and sale of data for advertising purposes. The United States Trade Representative considered this policy to be discriminatory against US companies and proposed retaliatory tariffs. Following the US reaction, France postponed the collection of the DST.

In the United Kingdom in April 2020, a 2% DST has been levied on revenues from social media platforms, internet search engines and online marketplaces.

In Austria in January 2020 a 5% DST has been levied on revenues from online advertising. This measure applied only to companies whose revenues exceed €750 millions worldwide and exceeding €25 millions in Austria.

Outside Europe, other countries have also adopted DST (e.g., India, Indonesia and Tunisia) or announced or show intention to adopt DST (e.g., Brazil, Kenya, Canada, Israel and New Zealand). On the contrary, Chili has rejected the proposal of a DST.

This experience has created potential rooms for retaliation, trade wars, tax avoidance and hence has highlighted the need of international coordination.

Over the last years the OECD and the European Commission have put forth proposals and started negotiations. An agreement was reached only in the second half of 2021.

Over the last years, the OECD has hosted negotiations with 139 countries to revise the international tax system and require that profits run by multinational enterprises are subject to taxation also in those countries where enterprises sell their products and services even without having a physical presence.

On 1 July 2021, the OECD Inclusive Framework issued the key principles defining the new taxation system for multinational companies.^11^ The agreement has been signed on 8 October 2021. The new agreement establishes two pillars. Pillar 1 states that business with an annual turnover exceeding EUR 20 billions and a margin of profit above 10% will be subject to taxation in those countries where customers are located. Pillar 2 establishes a minimum tax rate of 15% for multinational companies with an annual turnover exceeding EUR 750 millions.

New taxing rights for market countries at the expense of residence countries, along the lines of proposals discussed under Pillar 1 of the OECD-Inclusive Framework (IF) will change the geographic distribution of tax revenues paid by digital enterprises. Countries imposing low corporate tax and with investment hubs are likely to lose revenues as less profits will be shifted toward them. Conversely, those countries where multinational enterprises are not headquartered but have customers are likely to gain revenues from the reallocation.^12^

### Other Proposals

Some other alternatives to the robot tax are imposing a higher VAT tax on buying robot systems, or government's purchase of shares in companies and participation in dividends that can be redistributed to the population.

Recently, Saez and Zucman (2021) have proposed to introduce a new tax on corporations' stock shares for all companies with headquarter in G20 countries. This proposal stems from the idea that in the globalized world some companies may establish market power and raise enormous profits and wealth. Since stock ownership is highly concentrated in the hands of the richest, this tax on corporation stock shares would be progressive. To avoid liquidity issues, the tax could be paid by issuing new stock.

In a similar vein, Miles Kimball and Bloomberg writer Noah Smith suggest the creation a sovereign-wealth fund, split into many smaller funds, to avoid ownership concentration. Government could buy stocks and real estate using tax revenues and then distribute the profits to the society. In this way, governments would redistribute some of the profits arising from robotization.

Finally, another solution could be a wage subsidy for low-income workers. The most direct way is to cut payroll taxes, which overly burden low-paid workers. To fund social security, governments can use other sources, for instance increasing income taxes on the richest or a value-added tax. This is basically a shortcut to make human workers cheaper. However, while this solution reduces inequality in the short run, it may slow down productivity in the long run since it preserves unskilled labor employment which is less productive than robots. Therefore, in adopting this policy governments should balance trade-off effects in the short and long run (Berg et al., 2021).

## Conclusions

While there are several proposals on the table, the only concrete steps, although very timid, undertaken so far concern the robot tax and the digital tax. There are a few ideas defined as “robot tax”, but they vary significantly in design and magnitude. For example, the so-called robot tax in South Korea is a measure to reduce tax incentives for investment in automation rather than a tax on robots as proposed by Bill Gates. The idea of a robot tax as a way to levy companies directly on their use of robots and to apply those revenues toward a universal basic income is indeed philosophically appealing. However, it is overly unrealistic to expect that companies will pay for it through an income tax on their robots and AI networks.

Finally, possible widening inequalities caused by technological change may require different tax policies, depending on whether inequality is arising from skill-biased technological change or from the emergence of “superstar” firms in the digital economy. In the first case, to preserve employment and especially low-skilled workers, the robot tax could be a valid solution. However, it is also true that, as side effect, the robot tax can impede innovation. To avoid this side effect, the “design” of the robot tax is crucial. A solution could be to levy the robot tax just for a limited time, to preserve employment and have the necessary time to re-skill workers and provide them with the new skills and competencies requested on the market. The alternative, levying the robot tax as a lump-sum tax, may be not distorsive, but it will bear more on small businesses with high costs in terms of inequality. To preserve low-skill and low-paid employment, an alternative to the robot tax could be to provide wage subsidies for low-income workers. However, in choosing tax instruments governments should find the right balance between reducing inequality and preserving long-term productivity and growth. Wage subsidies for low-paid workers may be successful in preserving low-skill employment and reduce inequality in the short-run, but at cost of lower productivity in the long-run.

In the second case, when the main driver of inequality is the dichotomy between digital “superstar” firms and traditional business, the digital tax is a valid tool. There are no side effects arising from a digital tax *per se*. However, due the cross-border nature of digital businesses, digital taxation requires international coordination and multilateral action to avoid harmful retaliation and trade wars. Other solutions consist in redistributing profits from “superstars” to the society though the creation of a sovereign wealth fund or the introduction of a tax on corporation stock shares.

Not necessarily a tax option is preferable compared to the others and the discussed proposals are not mutually exclusive. Of course, policy-makers always have to keep in mind synergies between policy instruments.

To reduce inequality and achieve a more inclusive growth, tax policies should go hand in hand with other types of policies, such as education and training to guarantee that workers gain competencies demanded by the new digital economies, as well as competition policies to avoid concentration of market power in the hands of a limited number of “superstar” firms.

Within this debate, new points of discussion are emerging. Data are necessary for machine learning projects and predictive models which allow companies to provide better customer service, refine and personalize marketing and ultimately increase their profits. Users often disclose their personal data without being aware of how much information they are providing and how much digital firms monetize it. An interesting point of discussion which is recently arising is the opportunity to tax digital companies for profiting from users' personal data. In 2018 the European Commission has proposed to adopt tax measures on revenues created from activities where users play a major role in value creation. However, no further measures have been adopted at European level. Alternatively, if data can be treated as labor, users should be compensated for providing data. Since consumers have no bargaining power vis-à-vis digital firms, it is quite unrealistic that consumers can be compensated if they sell data individually. A solution could be the creation of “mediators of individual data” that would collect users' data and negotiate agreements with firms according to a transparent setting price mechanism (Lanier and Weyl, 2018). This field certainly deserves more analysis and research.

## Data Availability Statement

The original contributions presented in the study are included in the article/supplementary material, further inquiries can be directed to the corresponding author/s.

## Author Contributions

The author confirms being the sole contributor of this work and has approved it for publication.

## Conflict of Interest

RM was employed by the ILO.

## Publisher's Note

All claims expressed in this article are solely those of the authors and do not necessarily represent those of their affiliated organizations, or those of the publisher, the editors and the reviewers. Any product that may be evaluated in this article, or claim that may be made by its manufacturer, is not guaranteed or endorsed by the publisher.

## Footnotes

^1^The Future of Jobs Report 2018 (World Economic Forum, 2018).

^2^For a wider discussion of the literature on job implications of AI, we refer to Ernst et al. (2019).

^3^For a discussion on alternative options for taxing profits and rents generated by the collection and the process of users' data, see Aslam and Shah (2020).

^4^I refer the reader to “Big Data and its enclosure of the commons”, published in *Social Europe* on June 12, 2019.

^5^On this argument, Acemoglu and Restrepo (2018) wrote, “The vast majority of tax revenues are now derived from labour income, so firms avoid taxes by eliminating employees.” *New York Times* journalist Eduardo Porter wrote, “Machines don't incur payroll taxes, which are used to fund Social Security and Medicare. For every worker replaced by a robot, the employer saves on payroll taxes.”

^6^In OECD countries, in 2015 individual income taxes and social insurance taxes represented approximately 50% of all tax revenues. In the United States, the reliance on labor taxation is even more pronounced, with more than 60% of all tax revenue coming from individual income taxes or payroll taxes (see https://taxfoundation.org/publications/sources-of-government-revenue-in-the-oecd/).

^7^See Eduardo Porter, “Don't Fight the Robots, Tax Them”, N.Y. TIMES (Februaty 23, 2019).

^8^I refer to Oberson (2017) for more details.

^9^In some countries, some categories of goods or services are not subject to VAT (e.g. e-books, online courses), See Bunn et al. (2020), in particular Table 4.

^10^For further information, I refer the reader to Bunn et al. (2020).

^11^For more details we refer the reader to OECD/G20 Base Erosion Profit Shifting Project (2021). https://www.oecd.org/tax/beps/statement-on-a-two-pillar-solution-to-address-the-tax-challenges-arising-from-the-digitalisation-of-the-economy-july-2021.pdf and https://www.oecd.org/tax/beps/brochure-two-pillar-solution-to-address-the-tax-challenges-arising-from-the-digitalisation-of-the-economy-october-2021.pdf.

^12^For a discussion on Asia, see IMF (2021).
